# Establishing a composite endpoint for measuring the effectiveness of geriatric interventions based on older persons’ and informal caregivers’ preference weights: a vignette study

**DOI:** 10.1186/1471-2318-14-51

**Published:** 2014-04-18

**Authors:** Cynthia S Hofman, Peter Makai, Han Boter, Bianca M Buurman, Anton JM de Craen, Marcel GM Olde Rikkert, Rogier ART Donders, René JF Melis

**Affiliations:** 1 Department of Geriatric Medicine (HP 925), Radboud University Medical Center, PO Box 9101, 6500 HB Nijmegen, Netherlands; 2 Department for Health Evidence (HP133), Radboud University Medical Center, PO Box 9101, 6500 HB Nijmegen, Netherlands; 3 Department of Epidemiology (FA41), University of Groningen, University Medical Centre, Groningen, PO Box 30001, 9700 RB, Groningen, Netherlands; 4 Department of Internal Medicine, Section of Geriatric Medicine (F4-108), Academic Medical Center, PO Box 22660, 1100 DD Amsterdam, Netherlands; 5 Department of Gerontology and Geriatrics (C2-R), Leiden University Medical Centre, PO Box 9600, 2300 RC Leiden, Netherlands

**Keywords:** Composite endpoint, Preference-weighted, Elderly persons, Informal caregivers, Effectiveness, Geriatric interventions

## Abstract

**Background:**

*The Older Persons and Informal Caregivers Survey Minimal Dataset’s* (TOPICS-MDS) questionnaire which measures relevant outcomes for elderly people was successfully incorporated into over 60 research projects of the Dutch National Care for the Elderly Programme. A *composite endpoint* (CEP) for this instrument would be helpful to compare effectiveness of the various intervention projects. Therefore, our aim is to establish a CEP for the TOPICS-MDS questionnaire, based on the preferences of elderly persons and informal caregivers.

**Methods:**

A vignette study was conducted with 200 persons (124 elderly and 76 informal caregivers) as raters. The vignettes described eight TOPICS-MDS outcomes of older persons (morbidity, functional limitations, emotional well-being, pain experience, cognitive functioning, social functioning, self-perceived health and self-perceived quality of life) and the raters assessed the general well-being (GWB) of these vignette cases on a numeric rating scale (0–10). Mixed linear regression analyses were used to derive the preference weights of the TOPICS-MDS outcomes (dependent variable: GWB scores; fixed factors: the eight outcomes; unstandardized coefficients: preference weights).

**Results:**

The mixed regression model that combined the eight outcomes showed that the weights varied from 0.01 for social functioning to 0.16 for self-perceived health. A model that included “informal caregiver” showed that the interactions between this variable and each of the eight outcomes were not significant (p > 0.05).

**Conclusion:**

A preference-weighted CEP for TOPICS-MDS questionnaire was established based on the preferences of older persons and informal caregivers. With this CEP optimal comparing the effectiveness of interventions in older persons can be realized.

## Background

The number of elderly is increasing worldwide, due to increasing life-expectancy [1]. Ageing of our populations will have a major impact on the organization and delivery of health care, as healthcare systems have to meet the needs of geriatric patients, while the shortage of healthcare workers is likely to grow [2]. To restrain healthcare spending and improve the quality of care it is necessary to measure, report, and compare outcomes in healthcare delivery [3,4]. However, comparing intervention outcomes for elderly is a great challenge because their health states are complex with problems in multiple domains, e.g. morbidities and physical functioning, and interventions often target a broad range of domains [5]. A generic measurement instrument with a composite endpoint (CEP) would, therefore, be helpful to compare the effectiveness of different geriatric interventions.

With the increasing proportion of elderly and its impact on the organization and delivery of health care in mind, the Dutch Ministry of Health, Welfare, and Sport commissioned the National Care for the Elderly Programme (NCEP) with the aim to develop a more proactive, integrated healthcare system for older patients. Over 60 scientific projects were conducted under this programme [6]. To achieve standardized outcome measurements within the NCEP, The Older Persons and Informal Caregivers Survey Minimal DataSet (TOPICS-MDS) instrument was constructed and integrated into the research protocols [7]. TOPICS-MDS was developed by a small working group and includes validated instruments that are frequently used in older populations. Additionally, the instrument’s content and utility was evaluated by an independent multi-disciplinary panel with expertise in gerontology, epidemiology, biostatistics and health services research and a plain language expert was commissioned to revise the instrument for clarity and readability.

Although TOPICS-MDS is used to gather uniform data of the NCEP projects in a National Database (collecting dataset of over 32,000 elderly persons), there is currently no consensus on how to combine and weight the information from multiple outcome domains into a CEP. This means that the effectiveness of the projects can only be evaluated comparing the multiple individual domains separately and not the overall outcome [8]. Using a single TOPICS-MDS item or item subset to compare outcomes leads to confusion when competing projects demonstrate different patterns of effect, as the items or domains may not be equally important [9]. For example, it is difficult to decide which intervention is more effective if one intervention reduces the number of functional limitations and reduces pain sensation, while another improves social functioning en emotional well-being. Hence, for optimal comparison of the NCEP projects’ effectiveness a CEP that accounts for the relative importance of different outcomes is required.

In this study, we explore how multidimensional TOPICS-MDS outcomes from the Care receiver questionnaire can be weighted and combined into a CEP. The relative importance of the outcomes are reflected by preference weighting of TOPICS-MDS information compared with an anchor [10]. We opted for best and worst general well-being (GWB) as the anchor, because improving patients’ GWB is a goal all stakeholders share. Basically, GWB is a concept that covers a broad spectrum of health and it is influenced by various health outcome domains. Since the purpose of healthcare is to meet the needs of patients, our main focus should be on outcomes that matter to the patients [4,11,12]. However, as relatives of elderly persons often deliver informal care and serve as proxies, e.g. when the elderly person has a low cognitive status, we are interested in the relative importance of the items according to them as well [13]. Thus, the aim of this study is to examine the preference weights of elderly persons and informal caregivers and explore whether their preference weights differ.

## Methods

### Ethical approval

The Medical Ethics Committee of the Radboud University Medical Center formally stated that this study was exempt from ethical review (Radboud University Medical Center Ethical Committee review reference number: CMO: 2010/244).

### Study design

This study has three components that are similar to those described in the valuation study of Brazier, Roberts, and Deverill [14]. Firstly, TOPICS-MDS questionnaire for care receivers has been reduced in size and complexity. Secondly, a valuation study was conducted to derive the preference weights for the TOPICS-MDS outcomes. However, in contrast to the study of Brazier et al. we used a numeric rating scale to value the health states [14]. Thirdly, the results of the valuation study were used in a model to calculate the composite endpoint for the vignette cases.

### Vignette study

In our valuation study vignettes were being used. Over the last few years, the number of vignette studies increased in various fields of application, such as psychology, sociology, marketing, education and training, and clinical practice [15-19]. These kinds of studies are typically used to study the beliefs, values, or judgments of respondents [15]. Hence, they are useful to derive preference weights for single index values [14]. Vignettes are short descriptions of a person or a social situation which contain precise references to what are thought to be the most important factors in the decision- or judgment-making processes of respondents [16].

### Participants

A sample of 124 community dwelling elderly aged ≥ 65 years and 76 informal caregivers participated as raters. We used a rather broad definition of informal caregiver: “An informal caregiver provides voluntary and unpaid care on a structural basis to a care recipient with physical, mental or psychological limitations who is most often a relative, friend or neighbour. The provided care involves assisting the care receiver with tasks (s) he would do him-/herself in normal health” derived from the NCEP website [20]. In this study only informal caregivers who provided care to a care receiver aged ≥ 65 years were included. The participants were eligible if they mastered the Dutch language sufficiently. This was explored by the trained research assistants during first contact with the participants. When communication in Dutch was possible (asking questions regarding marital status, living arrangements, and family) the participants were included in the study.

The participants were recruited and the data was collected by four academic centres: Radboud University Medical Center, University Medical Centre Groningen (UMCG), Academic Medical Centre (AMC), and Leiden University Medical Centre (LUMC). These centres were spread over the Netherlands, and cover both urban and more rural parts of the country. To ensure a representative sample the participants were recruited in hospital outpatient clinics, general practitioner (GP) practices, nursing homes, day care facilities, and via the internet (recruitment messages were placed online). Written informed consent was obtained from each participant before the start of the vignette study.

## Material

In total 292 vignettes were constructed based on data of real persons (cases) derived from TOPICS-MDS National database. As the participants were asked to read the vignettes by themselves we used a large font size (14 points) and double spacing. In general, each vignette included 46 items and described elderly persons covering eight health domains: morbidity, functional limitations, emotional well-being, pain experience, cognitive functioning, social functioning, self-perceived health and self-perceived quality of life (QOL) and four demographic characteristics: gender, age, marital status, and living situation.

Table 1 gives an overview of the health domains, items per domain, and levels per item which were included in the vignettes and used in the analyses.

**Table 1:** The domains, items, and aggregated items included in the vignettes with the descriptives

**Outcome domains**	**Vignette items**	**Outcomes levels**	**Descriptives**
Morbidity (Local and national health monitor) [21]	Presence of: Dementia; Depression; Incontinence; Stroke, CVA or TIA; Hip fracture; Panic or anxiety disorder; Dizziness with falling; Vision disorder; Asthma; Osteoporosis; Diabetes; Arthritis; Heart failure; Form of cancer; Complaints due to benign enlarged prostate; Fracture other than hip fracture; Hearing disorder	Number of diseases present; *counting the number of health items “Present” Range: 0-17*	Mean: 3.5
			SD: 2.0
Functional limitations (modified KATZ-ADL Index) [22]	Needing help with: Brushing hair; Going to the toilet; Taking medication; Sitting down and getting up from chair; Getting dressed; Travelling; Handling finances; Grocery shopping; Walking about; Taking a bath or shower; Housekeeping; Preparing a meal; Eating; Using the telephone	Number of limitations in (I) ADL; *counting the number of physical functioning items “Help needed” Range: 0-15*	Mean: 3.0
			SD: 3.5
Emotional well-being (Rand-36, mental health subscale) [23]	Feeling down; Feeling blue; Feeling nervous; Feeling happy; Feeling calm	Raw mental health score; *Calculating the raw score of the five mental health items, each ranging from 1 to 6 Range: 5-30*	Mean: 10.4
			SD: 4.7
Pain experience (Single item EQ-5D + C) [24]	Pain experience	No	130 (44.7)
		Moderate	127 (43.6)
		Severe	34 (11.7)
Cognitive functioning (Single item EQ-5D + C) [24]	Cognitive problems	No	222 (77.1)
		Moderate	64 (22.2)
		Severe	2 (0.7)
Social functioning (Single item RAND-36) [23]	Social activities hampered by physical health or emotional problems	Never	217 (75.9)
		Rarely	19 (6.6)
		Sometimes	29 (9.1)
		Mostly	6 (2.1)
		Continuous	18 (6.3)
Self-perceived health (Single item, RAND-36) [23]	Self-perceived health in general	Excellent	18 (6.3)
		Very good	20 (7.0)
		Good	127 (44.3)
		Reasonable	108 (37.6)
		Poor	14 (4.8)
Satisfaction with quality of life (QOL) (Single item formed using phrasing similar to self-perceived health question, RAND-36) [23]	Self-perceived QOL in general	Excellent	26 (9.1)
		Very good	40 (14.0)
		Good	170 (59.4)
		Reasonable	41 (14.4)
		Poor	9 (3.1)

By using empirical data only vignettes with plausible health state combinations were constructed. The cases described in the vignettes had a mean age of 81.4 years (SD 5.72) and 58.6% (N = 171) was female. The majority of these cases were either married (42.8%, N = 125) or their partner was deceased (42.8%, N = 125), and 39.7% (N = 116) lived independently with someone, e.g. a partner or family member.

### Procedure

The vignette study was conducted in a familiar environment of the rater, e.g. in their own home or in a community centre in their living area. First, to collect the characteristics of the raters, we asked them to fill in the TOPICS-MDS themselves. Then, the vignette experiment started. After reading each vignette (see Additional file 1 for an example), participants were asked “How would you rate the general well-being of this person based on what you just read?”. A numeric rating scale was used to assess the general well-being of the cases according to the participants. The scale ranged from 0 to 10; with 0 representing the worst and 10 representing the best possible general well-being. The participants were allowed to use one decimal, this scale is in line with the Dutch grading system and is therefore well known to every Dutch person.

The vignette study began with two trial vignettes. These vignettes were the same for every participant and aimed to (1) help the participant understand the task; (2) determine whether the participant comprehend the Dutch language sufficiently to fulfil the task; and (3) give the participant an idea of the range among the vignettes with regard to how well or how poor the GWB of the cases could be. Comprehension of the Dutch language was sufficient when the participants were able to understand the text of the vignettes without asking for clarification. Understanding the range of the vignettes was achieved through presenting trial vignettes on both extremes of the range. After the two trial vignettes, the participants were asked to give scores to a selection of ten vignettes following the same procedure. The vignettes were randomly selected with Excel, making sure each vignette was not assessed by more than five elderly raters and not by more than three informal caregivers to ensure equal distribution of the vignettes.

In some cases two or more participants filled in the survey simultaneously, e.g. partners (two elderly raters) or pairs (an elderly rater and his or her informal caregiver). These participants were instructed to assess the vignettes independently, meaning they were not allowed to consult each other in any way. The interviewer checked participants’ adherence to this rule.

### Statistical analysis

#### Stage I

Mixed linear models were used to study the relationship between the eight outcomes from TOPICS-MDS care receiver questionnaire and raters’ GWB scores (0–10), to obtain the preference weights derived from scores given by the elderly raters and informal caregivers and to correct for clustering within raters (as each participant evaluated several vignettes) a random (participant dependent) intercept was included in the models.

First, a mixed model with random effects was constructed to obtain the preference weights for all raters, for both elderly raters and informal caregivers (N = 200). We used the GWB scores as dependent variable and the eight outcomes as independent variables (fixed factors). Then, we repeated the analysis with the variable “informal caregiver” (0/1; no/yes) as additional independent variable to explore the influence of the informal caregiver role on the preference weights using interaction effects. The participants who fulfilled the role as informal caregiver and were aged ≥ 65 years were included in the group informal caregivers.

#### Stage II

For the majority of the 292 vignette cases (95.5%, N = 279) we were able to calculate a TOPICS-CEP score (using the unstandardized coefficients found in stage I (Table 2) as preference weights) as they had no missing data points. Among these 279 cases 86.3% (N = 241) had rated their own GWB.

**Table 2:** Results of mixed linear analyses with as dependent variable the GWB scores given by all raters

	**Older persons and informal caregivers (N = 200)**				
	**Unstandardized coefficient**	**Standardized coefficient**		**Confidence interval for B**	
	**B**	**Beta**	**P-value**	**Lower limit**	**Upper limit**
Intercept	9.03	1.54	0.00**	8.84	9.22
Morbidities	−0.14	−0.10	0.00**	−0.15	−0.12
Functional limitations	−0.12	−0.09	0.00**	−0.13	−0.11
Emotional well-being	−0.04	−0.03	0.00**	0.13	0.23
Pain experience	−0.04	−0.03	0.17	−0.11	0.02
Cognitive functioning	−0.13	−0.10	0.00*	−0.22	−0.04
Social functioning	−0.01	−0.01	0.63	−0.04	0.02
Self-perceived health	−0.16	−0.12	0.00**	−0.20	−0.12
Self-perceived QoL	−0.03	−0.02	0.29	−0.07	0.02

Note 1: The unstandardized coefficients estimate the increase in the dependent variable (general well-being scores) per unit increase of the continuous predictors.

Note 2: The unstandardized coefficients are the preference weights of the components included in the composite endpoint.

*p < 0.05 **p < 0.001.

Differences in mean TOPICS-CEP scores between sexes and between age groups were explored using T-test and ANOVA, respectively. The same was done for the differences in mean self-assessment scores. Differences between the calculated TOPICS-CEP scores and the self-assessment scores were examined using a paired sample T-test and Pearson’s correlation.

## Results

### Raters

The participants included in the group elderly raters (N = 124) had a mean age of 78.3 years (SD 6.70) and 62.9% (N = 78) was female. The majority of these raters were married (59.7%, N = 74) and 60.5% (N = 75) lived independently with someone, e.g. their spouse or a relative. The elderly raters gave their own GWB a mean score of 7.7 (SD 0.92).

The 76 informal caregivers who participated in this study had a mean age of 63.0 years (SD 12.14), 72.4% (N = 55) was female, and 92.1% (N = 70) took care of a family member. The informal caregivers gave their own GWB a mean score of 7.2 (SD 1.15).

### Completion rates

There were 2400 numerical rating scale valuations completed by the participants out of the 2400 possible (124 × 12 for elderly raters and 76 × 12 for informal caregivers). All 200 participants were capable to read the vignettes themselves and language comprehension was not an issue.

#### Stage I

The linear mixed regression model that combined the eight outcomes showed that *p-value* of the outcomes: morbidities, limitations in daily functioning, emotional well-being, cognitive functioning, and self-perceived health was smaller than 0.05 (Table 2).

The linear mixed regression model that combined the eight outcome and the additional variable “informal caregiver” showed that the *p-value* of the outcomes: morbidity, functional limitations, emotional well-being, cognitive functioning, and self-perceived health was smaller than 0.05. In addition, the interactions between the “informal caregiver” variable and each of the domains were not significant (p > 0.05).

Examining the residuals we found no large departures from normality nor evidence for the presence of outliers. Based on the narrow confidence intervals multicollinearity between the outcome domains of the CEP is unlikely.

#### Stage II

**Figure 1:**
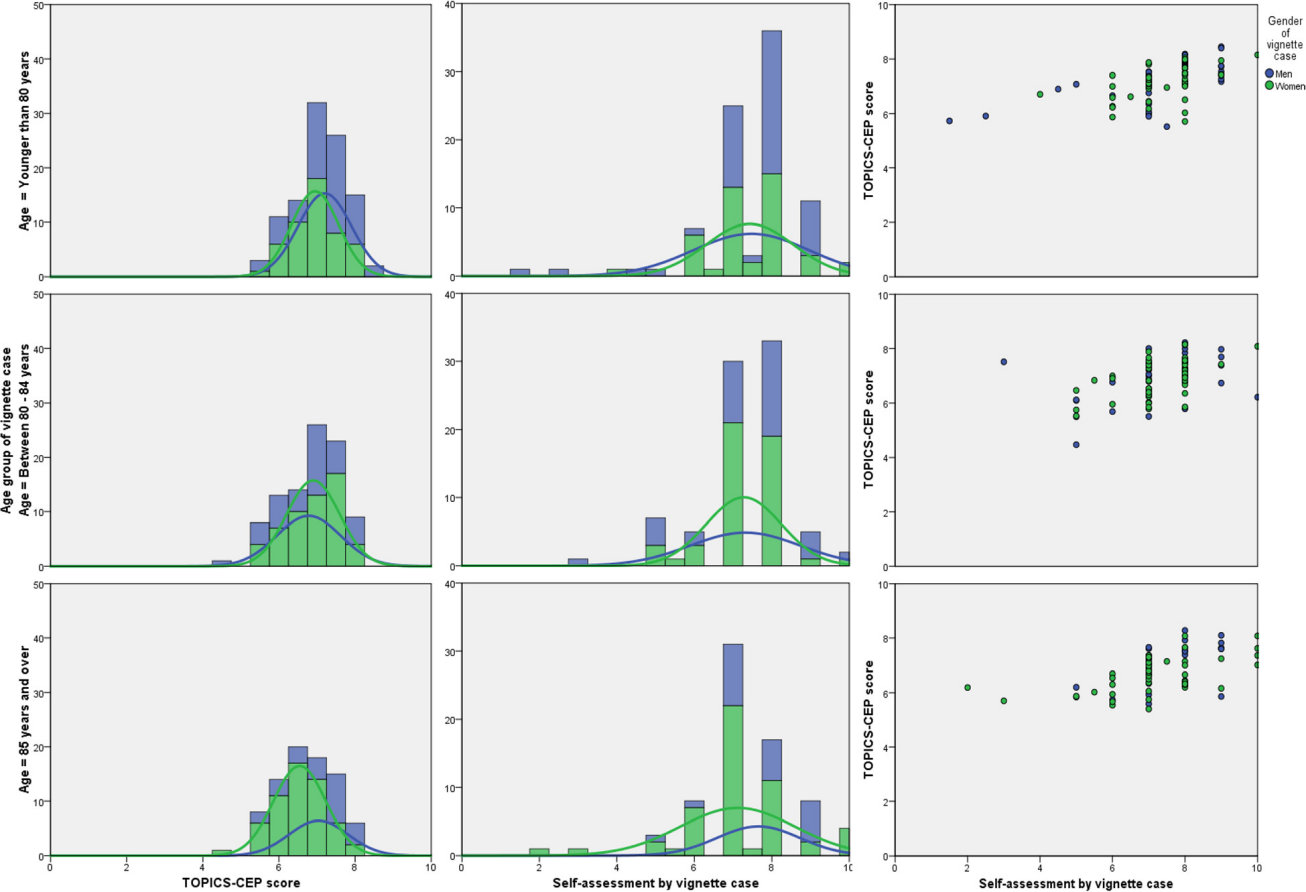
**Frequency distribution and correlation matrices for men (blue) and women (green) of TOPICS-CEP and self-assessment scores of the case vignettes by age groups (N = 241).** Overall, the self-assessment scores had a broader range compared to TOPICS-CEP scores. The correlation matrices indicate moderate correlation between the two scores for all age groups. Pearson correlation test on whole group (r = 0.52, p = 0.00).

Among the 282 of 292 vignette cases for whom a TOPICS-CEP could be established and who rated their own GWB, the minimum TOPICS-CEP score calculated was 4.72 and the maximum score was 8.45 [**Mean (±SD):** 6.95 (0.73)]. The overall distribution of the TOPICS-CEP scores was tailed to the left (not shown). The distribution of the TOPICS-CEP scores was more normalized within the age group aged *at least 85 years* than within the younger age groups (Figure 1). Mean TOPICS-CEP scores (±SD) significantly differed across sex and between age groups [**Men:** 7.10 (0.76); **Women:** 6.84 (0.67); p = 0.00] [**<80:** 7.15 (0.65); **80**–**84:** 6.90 (0.75); **≥85:** 6.74 (0.75); p = 0.00].

Of the 249 cases who rated their own GWB the majority gave their own GBW a score of 7.0 (33.6%) or 8.0 (35.7%) (Figure 1). Mean self-assessment scores (±SD) did not significantly differed across sex and between age groups [**Men:** 7.46 (1.40); **Women:** 7.26 (1.22); p = 0.25] [**<80:** 7.45 (1.33); **80**–**84:** 7.29 (1.18); **≥85:** 7.28 (1.38); p = 0.63].

Compared to TOPICS-CEP scores, the self-assessment scores had a broader range and a significantly higher mean [**Range:** 1.50-10.0; **Mean difference (±SD):** 0.34 (1.10), p = 0.00]. The two scores were moderately correlated (r = 0.52, p = 0.00).

## Discussion

Our primary findings support that a CEP for *TOPICS-MDS Care receiver questionnaire* can be established based on the preference weights of both elderly persons and informal caregivers, which were derived by means of our vignette study. The narrow confidence intervals of our estimated parameters suggest that there was enough information present in the dataset, hence, that the sample size was large enough. Our secondary analysis indicates that using a CEP that can be calculated based on assessments from patients (e.g. by means of a questionnaire) is related to GWB, yet measures a different concept as the correlation is of medium strength.

In contrast to previous research, elderly persons and informal caregiver (or family members) share the same preferences when it comes to the assessment of a subjective measure such as GWB [25-28]. Perhaps, the discrepancy between our findings and findings in other studies can be explained by the fact that in our study there was no personal relationship between the informal caregiver and elderly patient (cases described in the vignettes) that could influence the assessment made, e.g. response shift bias or caregiver burden [26,29-31]. We asked elderly persons and informal caregivers to assess the GWB of neutral cases, while in other studies elderly persons were asked to assess their own GWB and informal caregivers were asked to assess the GWB of their loved ones.

Our results and implications need to be interpreted in light of several limitations. First, the vignettes we used in this study were based on empirical data derived from the TOPICS-MDS National Database, which means that some combinations of the outcome domains were not represented, e.g. a case with dementia, dizziness with falling, hip fracture and fracture other than hip fracture who do not have any functional limitations. However, by using empirical data only vignettes with plausible health state combinations were constructed. Second, the distribution of marital status and living arrangement characteristics over the participants are similar to those over the Dutch population (≥65 years) [32]. However, in our study the elderly raters had a mean age of 78.3 years and 62.9% of the sample was female, while the mean age of the Dutch elderly population is 74.3 years and 56% of this population is female [32]. Hence, women and elderly aged 80 years and over are overrepresented in our sample. Previous research has shown individual variation in health state preferences influenced by gender and age [33,34]. Therefore, we will explore the influence of our raters’ characteristics on the TOPICS-CEP’s preference weights in our next study. Third, even though the most important health domains from *TOPICS –MDS Care receiver questionnaire* were included in the CEP there may be aspects that influence the general well-being of elderly that are not included in the questionnaire and the CEP, such as isolation and loneliness.

The benefits of using TOPICS-MDS and its’ CEP are that a range of important endpoints will be collected and incorporated in a single metric, which can index the overall impact of interventions according to elderly persons and informal caregivers in a standardized way and reduce sample size requirements. Hence, establishing the value of interventions will be easier and more objective. Similar to other composite endpoints, such as the Disease Activity Score in rheumatology, the use of TOPICS-CEP may improve analysis of clinical trials and it may even be applicable to clinical care [35,36].

For future research we suggest to explore the responsiveness of the established CEP and its prognostic value. Also, we advise to compare the preference weights of older persons and informal caregivers derived in this study with those of healthcare providers.

## Conclusions

TOPICS-MDS has been successfully incorporated into all NCEP research projects. Until now, the effectiveness of the projects could only be compared per item, item subset, or comparing multiple endpoints. With the establishment of TOPICS-CEP for the care receiver questionnaire that accounts for the relative importance of different outcomes based on the preferences of elderly persons and informal caregivers, optimal comparison of NCEP project’s effectiveness can be realized. A syntax to calculate the TOPICS-CEP score will be available on the TOPICS-MDS website in the latter half of 2013 [7].

Besides NCEP projects, other projects in the geriatric field can use the TOPICS-MDS to collect research data and the TOPICS-CEP allowing standardized assessment of patient outcomes reflecting the preferences of elderly persons and informal caregivers [7].

## Abbreviations

CEP: Composite endpoint; GWB: General well-being; NCEP: National Care for the Elderly Programme; TOPICS-MDS: The older persons and informal caregivers survey minimal dataset; TOPICS-CEP: The older persons and informal caregivers survey composite endpoint.

## Competing interests

The authors declare that they have no competing interest.

## Authors’ contributions

All authors were involved in the design and conceptualization of the study and in the acquisition of the subjects and data. AD and CH performed the statistical analyses. Together with PM, RM and MOR they interpreted the data and drafted the manuscript. All authors read and approved the final manuscript.

## Pre-publication history

The pre-publication history for this paper can be accessed here:

http://www.biomedcentral.com/1471-2318/14/51/prepub

## Supplementary Material

Additional file 1 **Example vignette.** The additional file is an example vignette. Click here for file

## References

[B1] Humphreys G The health-care challenges posed by population ageing Bull World Health Organ 2012 14 2 82 83 22423156 10.2471/BLT.12.020212 PMC3302561

[B2] How can health systems respond to population ageing? 2009 [http://www.euro.who.int/__data/assets/pdf_file/0004/64966/E92560.pdf]

[B3] Basu A Philipson TJ The impact of comparative effectiveness research on health and health care spending 2010 Cambridge: Mass. National Bureau of Economic Research 10.1016/j.jhealeco.2011.05.012 PMC3242481 21696840

[B4] Porter ME What is value in health care? N Engl J Med 2010 14 26 2477 2481 10.1056/NEJMp1011024 21142528

[B5] Fried LP Ferrucci L Darer J Williamson JD Anderson G Untangling the concepts of disability, frailty, and comorbidity: implications for improved targeting and care J Gerontol A Biol Sci Med Sci 2004 14 3 255 263 10.1093/gerona/59.3.M255 15031310

[B6] Lutomski JE Baars MA Schalk BW Boter H Buurman BM den Elzen WP Jansen AP Kempen GI Steunenberg B Steyerberg EW Olde Rikkert MG Melis RJ Topics-Mds Consortium The Development of the Older Persons and Informal Caregivers Survey Minimum DataSet (TOPICS-MDS): A Large-Scale Data Sharing Initiative PLoS One 2013 14 12 e81673 10.1371/journal.pone.0081673 24324716 PMC3852259

[B7] TOPICS-MDS - Supporting documentation [http://topics-mds.nl/wordpress/?page_id=34]

[B8] Neuhauser M How to deal with multiple endpoints in clinical trials Fund Clinical Pharma 2006 14 6 515 523 10.1111/j.1472-8206.2006.00437.x 17109645

[B9] Veit CT Ware JE Kane RL, Kane RA Measuring health state and health care outcomes: Issues and recommendations Values and Long Term Care 1982 Lexington MA: Lexington Books 233 259

[B10] Sackett DL Torrance GW The utility of different health states as perceived by the general public J Chron Dis 1978 14 11 697 704 10.1016/0021-9681(78)90072-3 730825

[B11] Gerteis M Picker/Commonwealth Program for Patient-Centered Care Through the patient's eyes: understanding and promoting patient-centered care 1993 1 San Francisco: Jossey-Bass

[B12] Say RE Thomson R The importance of patient preferences in treatment decisions–challenges for doctors BMJ 2003 14 7414 542 545 10.1136/bmj.327.7414.542 12958116 PMC192849

[B13] Van Houtven CH Norton EC Informal care and health care use of older adults J Health Econ 2004 14 6 1159 1180 10.1016/j.jhealeco.2004.04.008 15556241

[B14] Brazier J Roberts J Deverill M The estimation of a preference-based measure of health from the SF-36 J Health Econ 2002 14 2 271 292 10.1016/S0167-6296(01)00130-8 11939242

[B15] Atzmuller C Steiner PM Experimental Vignette Studies in Survey Research Methodol-Eur 2010 14 3 128 138

[B16] Alexander CS Becker HJ The use of vignettes in survey research Public Opin Q 1978 14 1 93 104 10.1086/268432

[B17] Ludwick R Zeller RA The factorial survey: an experimental method to replicate real world problems Nurs Res 2001 14 2 129 133 10.1097/00006199-200103000-00009 11302293

[B18] Farshad M Gerber C Szucs T Meyer DC Determining utility values in patients with anterior cruciate ligament tears using clinical scoring systems BMC Health Serv Res 2011 14 182 10.1186/1472-6963-11-182 21813026 PMC3160876

[B19] Muller-Engelmann M Krones T Keller H Donner-Banzhoff N Decision making preferences in the medical encounter–a factorial survey design BMC Health Serv Res 2008 14 260 10.1186/1472-6963-8-260 19091091 PMC2628895

[B20] Definitie mantelzorg (In Dutch only) [http://www.nationaalprogrammaouderenzorg.nl/fileadmin/www.npoz.nl/documenten/toolkits/mantelzorgdefinitie_def.pdf]

[B21] Lokale en nationale monitor gezondheid (In Dutch only) [https://www.monitorgezondheid.nl/gezondheidindicatoren.aspx]

[B22] Weinberger M Samsa GP Schmader K Greenberg SM Carr DB Wildman DS Comparing proxy and patients’ perceptions of patients’ functional status: results from an outpatient geriatric clinic J Am Geriatr Soc 1992 14 6 585 588 1587975 10.1111/j.1532-5415.1992.tb02107.x

[B23] VanderZee KI Sanderman R Heyink JW de Haes H Psychometric qualities of the RAND 36-Item Health Survey 1.0: a multidimensional measure of general health status Int J Behav Med 1996 14 2 104 122 10.1207/s15327558ijbm0302_2 16250758

[B24] Krabbe PF Stouthard ME Essink-Bot ML Bonsel GJ The effect of adding a cognitive dimension to the Euro Qol multiattribute health-status classification system J Clin Epidemiol 1999 14 4 293 301 10.1016/S0895-4356(98)00163-2 10235169

[B25] Gomez-Gallego M Gomez-Amor J Gomez-Garcia J Determinants of quality of life in Alzheimer's disease: perspective of patients, informal caregivers, and professional caregivers Int Psychogeriatr 2012 14 11 1805 1815 10.1017/S1041610212001081 22697366

[B26] Huang HL Chang MY Tang JS Chiu YC Weng LC Determinants of the discrepancy in patient- and caregiver-rated quality of life for persons with dementia J Clin Nurs 2009 14 22 3107 3118 10.1111/j.1365-2702.2008.02537.x 19207789

[B27] Crespo M Bernaldo de Quiros M Gomez MM Hornillos C Quality of life of nursing home residents with dementia: a comparison of perspectives of residents, family, and staff Gerontol 2012 14 1 56 65 10.1093/geront/gnr080 21903614

[B28] Moyle W Murfield JE Griffiths SG Venturato L Assessing quality of life of older people with dementia: a comparison of quantitative self-report and proxy accounts J Adv Nurs 2012 14 10 2237 2246 10.1111/j.1365-2648.2011.05912.x 22211637

[B29] Schwartz CE Sprangers MA Methodological approaches for assessing response shift in longitudinal health-related quality-of-life research Soc Sci Med 1999 14 11 1531 1548 10.1016/S0277-9536(99)00047-7 10400255

[B30] Rapkin BD Schwartz CE Toward a theoretical model of quality-of-life appraisal: Implications of findings from studies of response shift Health Qual Life Outcomes 2004 14 14 10.1186/1477-7525-2-14 15023229 PMC408464

[B31] Neumann PJ Araki SS Gutterman EM The use of proxy respondents in studies of older adults: lessons, challenges, and opportunities J Am Geriatr Soc 2000 14 12 1646 1654 11129756 10.1111/j.1532-5415.2000.tb03877.x

[B32] Bevolking; geslacht, leeftijd en burgerlijke staat (In Dutch only) [http://statline.cbs.nl/StatWeb/selection/?DM=SLNL&PA=7461BEV&VW=T]

[B33] Dolan P Effect of age on health state valuations J Health Serv Res Policy 2000 14 1 17 21 10787582 10.1177/135581960000500106

[B34] Wittenberg E Halpern E Divi N Prosser LA Araki SS Weeks JC The effect of age, race and gender on preference scores for hypothetical health states Qual Life Res 2006 14 4 645 653 10.1007/s11136-005-3514-3 16688497

[B35] van der Heijde DM van't Hof MA van Riel PL van Leeuwen MA van Rijswijk MH van de Putte LB Validity of single variables and composite indices for measuring disease activity in rheumatoid arthritis Ann Rheum Dis 1992 14 2 177 181 10.1136/ard.51.2.177 1550400 PMC1005654

[B36] Fuchs HA The Use of the Disease-Activity Score in the Analysis of Clinical-Trials in Rheumatoid-Arthritis Br J Rheumatol 1993 14 11 1863 1866 7905924

